# Measuring students’ learning progressions in energy using cognitive diagnostic models

**DOI:** 10.3389/fpsyg.2022.892884

**Published:** 2022-08-09

**Authors:** Shuqi Zhou, Anne Traynor

**Affiliations:** ^1^College of Foreign Languages, Donghua University, Shanghai, China; ^2^Department of Educational Studies, Purdue University, West Lafayette, IN, United States

**Keywords:** cognitive diagnostic model, energy concept, learning progression, mastery pattern, instructional sensitivity

## Abstract

This study applied cognitive diagnostic models to assess students’ learning progressions in energy. A Q-matrix (i.e., an item attribute alignment table) was proposed based on existing literature about learning progressions of energy in the physical science domain and the Trends in International Mathematics and Science Study (TIMSS) assessment framework. The Q-matrix was validated by expert review and real data analysis. Then, the deterministic inputs, noisy ‘and’ gate (DINA) model with hierarchical relations was applied to data from three jurisdictions that had stable, defined science curricula (i.e., Australia, Hong Kong, and Ontario). The results suggested that the hypothesized learning progression was consistent with the observed progression in understanding the energy concept. We also found similarities in students’ attribute mastery across the three jurisdictions. In addition, we examined the instructional sensitivity of the selected item. We discuss several curriculum-related issues and student misconceptions that may affect students’ learning progressions and mastery patterns in different regions of the world.

## Introduction

Students’ domain-specific concept knowledge has received substantial attention from researchers in science education (Liu and McKeough, 2005). Previous research shows that many students have not mastered an understanding of energy as envisioned in policy documents (Neumann et al., 2013). However, understanding energy is important, since energy concepts are scientifically and academically related to many social, environmental, and technological applications (Chen et al., 2014). Although there are extensive studies probing students’ understanding of energy (e.g., Liu and McKeough, 2005; Lee and Liu, 2010; Duit, 2014; Lacy et al., 2014), most studies use interviews (e.g., Jin and Wei, 2014; Lacy et al., 2014) and Rasch’s (1966) item response model (Liu and McKeough, 2005; Neumann et al., 2013). Rasch’s (1966) item response model applies a restrictive model assuming all items are equally discriminating indicators of students’ energy understanding. However, item discrimination often varies in practice. More importantly, the single score attained from any unidimensional item response model which includes Rasch model cannot provide evidence of students’ specific strengths or weaknesses, which are needed to identify effective classroom instructional practices. The interview studies are limited by the small sample sizes of participants, which affects their generalizability.

Students’ incorrect responses during problem-solving can be caused by weaknesses in multiple, distinct underlying skill attributes (e.g., Brown and VanLehn, 1980; Tatsuoka, 1983). Cognitive diagnostic models (CDMs) can uncover students’ mastery of multiple fine-grained skill attributes or problem-solving processes. CDMs are special cases of latent class models that characterize the relationship of observable data to a set of categorical latent ability attributes (typically dichotomous; Templin and Henson, 2006). CDMs can diagnose students’ performance on a set of multiple discrete skills and provide formative diagnostic information to inform instruction and learning based on students’ mastery or non-mastery of these fine-grained skills (Leighton and Gierl, 2007). Attributes and Q-matrix are two important terms in CDMs. Conceptually, the term attributes refer to “skills, dispositions, or any other constructs that are related to behavioral procedures or cognitive processes that a learner must engage in to solve an assessment item” (Carragher et al., 2019). Psychometrically, attributes refer to unobserved (latent) variables in a statistical model, which are measured by assessment items and encoded in a Q-matrix (Carragher et al., 2019), where Q-matrixis the loading matrix or pattern matrix that shows the relation of items and latent variables (Rupp et al., 2010). Based on students’ performance in the test, CDMs can measure students’ mastery patterns of the attributes needed in the test and thus provide diagnostic information to teachers. The empirical research findings showed promise that teachers and students can use feedback from these models to decide next steps in learning in different learning domains, such as reading (e.g., Kim, 2015), listening (e.g., Min and He, 2022), writing (e.g., Xie, 2017), and mathematics (e.g., Wu, 2019). Tang and Zhan (2021) also showed that CDM promotes students’ learning. However, CDMs have not been applied to the energy domain to characterize or support students’ learning. CDMs can help to diagnose students’ mastery of specific energy concepts, which could be useful to validate learning progressions (an ordered description of students’ understanding of a particular concept) that have been proposed in the literature since the aggregated mastery probabilities for each attribute in the CDMs are informative for the learning progressions (Briggs and Alonzo, 2012).

The aim of the study is to use CDM to analyze specific knowledge structures and processing skills involved in learning energy to provide information about students’ cognitive strengths and weaknesses. The specific aims are (a) to measure systematic patterns of students’ knowledge mastery and misunderstandings of energy and (b) to gain a better understanding of students’ learning progressions through energy concepts. The study will use CDMs to identify students’ knowledge mastery and misunderstanding patterns through the hypothesized learning progression. Since students’ opportunity to learn is an essential factor contributing to their learning outcomes (e.g., Törnroos, 2005), the study will also examine how the intended curriculum may influence students’ understanding of energy across different jurisdictions.

Based on previous research (Neumann et al., 2013; Lacy et al., 2014), this study hypothesizes that students understand energy by four hierarchical concepts: (1) forms of energy; (2) transfer and transformations of energy; (3) dissipation and degradation of energy; and (4) conservation of energy. The study will use data from a fourth-grade physical science assessment to address the following major questions:

1. To what extent does the hypothesized learning progression match students’ observed progressions in understanding the energy concept, based on the results of the cognitive diagnostic model?
2. What similarities in students’ knowledge mastery patterns are evident for different jurisdictions?
3. How does the intended curriculum relate to students’ understanding of energy across different jurisdictions?

## Learning progressions of energy

Learning progressions (LPs) are descriptions of increasingly sophisticated levels of thinking about or understanding a topic [National Research Council (NRC), 2007]. In the past two decades, as a core science concept, energy has received a lot of attention in the research on LPs across different grades or grade bands (e.g., Liu and McKeough, 2005; Lee and Liu, 2010; Neumann et al., 2013; Yao et al., 2017). These studies aim to develop corresponding assessments, examine students’ progression in understanding energy, and improve instruction and curriculum related to energy topics. Similar to approaches to developing learning progressions in other concepts in science, the development of LPs on energy mainly has used interviews (Dawson-Tunik, 2006; Lacy et al., 2014) and Rasch-type partial credit models (Lee and Liu, 2010; Neumann et al., 2013; Yao et al., 2017; Herrmann-Abell et al., 2018). The studied grades have ranged from third grade to twelfth grade. These studies include not only small samples but also large-scale samples, such as participants in the TIMSS (Liu and McKeough, 2005; Lee and Liu, 2010). Though studies may use different terms to refer to the same concepts, most of these studies propose LPs of energy from four strands: energy sources and forms, transfer and transformation, degradation, and conservation. We will introduce the studies specifically as follow.

Liu and McKeough’s (2005) results supported their hypothesized five levels of an energy concept sequence (i.e., activity/work, source/form, transfer, degradation, and conservation). Their study also showed that third-and fourth-grade students can develop an understanding of the first two levels, that is, energy does work, and sources or forms of energy. They also concluded that energy degradation should be an important component for understanding energy conservation (Liu and McKeough, 2005). Herrmann-Abell et al. (2018) examined Grade 6 to college students’ understanding of energy transformation, energy transfer, and conservation of energy using Rasch analysis. Their study supported that knowledge of forms of energy was important for students to successfully answer questions about energy transformation. They found the idea of conservation of energy was much more difficult than the ideas of energy transformation and energy transfer to students. They concluded that it was easier for students to know general principles than to apply them in real life.

Neumann et al. (2013) explored four hierarchical energy topics: forms, transfer, degradation, and conservation, each of which was conceptualized as having four hierarchical levels of complexity: facts, mappings, relations, and concepts. They confirmed a general progression of the four levels for energy conceptions (forms and sources, transfer and transformation, dissipation, and conservation), but they did not confirm the distinct levels of these conceptions. Their Rasch analysis and analysis of variance (ANOVA) suggested that students may develop an understanding of energy transfer and transformation in parallel with an understanding of energy degradation. Following Neumann et al.’s (2013) approach, Yao et al.’s (2017) study examined eighth- to twelfth-grade students’ developing understanding of energy in mainland China. Although their study followed the same sequence of four ideas about energy as Neumann et al.’s (2013) study, their Rasch analysis results did not support the hypothesis that students actually progress along this sequence in their understanding of energy. Their findings showed that although “energy forms” are a foundational idea for developing a deeper understanding of energy, other ideas may not necessarily be developed in a distinct sequence (Yao et al., 2017).

To allow students to accomplish understanding by the end of the elementary grades, Lacy et al. (2014) proposed a detailed learning progression for energy from four strands, focusing on grades 3–5: forms of energy, transfer and transformations, dissipation and degradation, and conservation. Their proposed learning progression was established on the “aligned development of a network of interconnected and interdependent foundational ideas” (p. 265). Their proposed progression was also based on students’ intuitive ideas. The progression also takes students’ misinterpretations and hurdles in previous research into account. Their exploratory interviews and teaching interventions have supported that relevant instruction could increasingly enhance, transform, and integrate students’ knowledge toward a scientific understanding of energy. Since this study will focus on Grade 4 students, we will hypothesize that students understand energy from four hierarchical concepts from the findings of Lacy et al.’s (2014) research: (1) forms of energy; (2) transfer and transformations of energy; (3) dissipation and degradation of energy; and (4) conservation of energy.

### Instructional sensitivity and science curriculum of primary schools across three jurisdictions

Instructional sensitivity is “the degree to which students’ performances on a test accurately reflect the quality of instruction specifically provided to promote students’ mastery of what is being assessed” (Popham, 1971, p. 1). Instructional sensitivity is a concept related to the opportunity to learn. Through instructional sensitivity, we can see how the instructional opportunity can influence students’ attribute mastery from the cognitive diagnostic model. As part of instructional opportunity, instruction and curriculum could influence students’ learning progressions (Duncan and Hmelo-Silver, 2009). Learning progressions cannot develop without scaffolded instruction or curriculum (Duncan and Hmelo-Silver, 2009). Different written curricula (i.e., the intended curriculum) could be one possible reason leading to the difference in students’ performance (Schmidt et al., 2001). In this study, we will focus on how the written science curriculum as an instructional opportunity relates to students’ attribute mastery, performance, and learning progressions.

Written curricula have been revealed to influence students’ performance across countries (Schmidt et al., 2001; Ramírez, 2006). Different countries and regions have different science curricula. The detailed expectations specified in the curriculum may also vary by country or region. We will introduce the science curriculum of the three jurisdictions (i.e., Australia, Hong Kong, and Ontario) that will be included in this study. Australia, Hong Kong, and Ontario are chosen since their curriculum has changed or been updated before 2011, and they also have clear science curriculum descriptions. These three jurisdictions participated in the TIMSS 2011 assessment, which included items measuring understanding of energy and item-level curriculum coverage information. In addition, Ontario is chosen as one of six benchmarking participants in 2011 TIMSS.

## Materials and methods

### Data

This study used TIMSS student achievement test data and curriculum data from Grade 4 and Year 2011 [International Association for the Evaluation of Educational Achievement (IEA), 2013]. TIMSS applies a two-stage random sample design: In the first stage, a sample of schools was drawn; in the second stage, one or more intact classes of students were selected from sampled schools (Martin et al., 2016). TIMSS 2011 assembled achievement test items in 14 booklets. Each item appeared in two booklets, and each student completed one booklet (Martin et al., 2016). Thus, there are designed missing responses of each item (about 85% designed missingness for each item of the selected data). The TIMSS datasets are suitable for the current investigation because (1) they provide reliable data on students’ science achievement, including performance on the energy topic, which is the main focus of the study; and (2) it also provides curriculum data from countries, which allows us to analyze and compare how the science curriculum may relate to students’ understanding of energy across countries. In 2011, Australia had 6,146 students, Ontario had 4,568 students, and Hong Kong had 3,957 students who participated in TIMSS.

### Variables

#### Student level variables

We focused on achievement test item variables assessing each student’s knowledge mastery of energy topics under the physical science domain in the year 2011. The cognitive domain of each item is specified in the assessment’s framework. Specific item IDs are listed in Supplementary Appendix Table A.1. There are 28 items in total. In total, 12 items only had two score categories, and all other items had more than two score categories. It should be noted here that items have multiple types of correct answers and/or multiple types of incorrect answers, but do not have partially correct answers. The items with more than two score categories were classified into two categories (correct will be coded as 1 and incorrect will be coded as 0) in the analysis.

#### Country-level variables

Country-level variables came from TIMSS test-curriculum matching analysis (TCMA). TCMA was conducted to investigate “the appropriateness of the TIMSS mathematics and science assessments for the fourth and eighth grade students in the participating countries” (Foy et al., 2013, p. 102). Binary coding indicated whether items in the assessment were included in the national curriculum, or not, for a particular participating country.

### Analysis

The data analysis was divided into four steps, and we followed the Q-matrix validation procedure of Mirzaei et al. (2020) while we improve their procedure by validating through different data (see detail below). First, after experts reviewed the proposed Q-matrix and we revised the Q-matrix (these would be introduced in the following sections), we used CDM to analyze and validated our revised Q-matrix. We randomly drew half of the data within each jurisdiction and combined those into one dataset for the first validation of the Q-matrix. We combined the rest of each jurisdiction’s data for the second validation. We revised the Q-matrix according to the first analysis result, again referring to the expert review information. Then, we used the second half of the combined dataset to do the second validation analysis. We conducted the validation analysis using the R software CDM package (Robitzsch et al., 2022). Since most of the items (91.66%) only measure one attribute in this study (see Supplementary Appendix Table A.2), the results of CDM analysis are expected to be similar across different models. Thus, we used the parsimonious and interpretable “deterministic inputs, noisy, ‘and’ gate” (DINA; Junker and Sijtsma, 2001) model, with results obtained by weighted maximum likelihood estimation and adding sampling weights to deal with specific sampling features and missingness in the survey data. Maximum likelihood estimation allows us to “estimate a set of parameters that maximize the probability of getting the data that was observed” (Newman, 2003, p. 332), and it is an effective way to treat missingness on outcome variables. Adding sampling weights to the analysis allows the sample results to reconstruct those that would be obtained if it was a random draw from the total population and leads to accurate population parameter estimates (Friedman, 2013).

We also compared the unrestricted DINA model and a more general model G-DINA using the likelihood ratio test (Robitzsch et al., 2022). The unrestricted DINA model was not significantly worse fitting than the G-DINA model (*χ*^2^ = 9.26, *df* = 4, *p* = 0.05486). Thus, we used the unrestricted DINA model for parsimony. We also tested the DINA model by specifying the hierarchical relations between attributes according to our hypothetical learning progression, that is, the attributes from the first strand of the learning progression should be mastered before those from the second strand. Comparing it to the unrestricted DINA model through likelihood ratio tests (*χ*^2^ = −0.07053, *df* = 4, *p* = 1), the *χ*^2^ is negative, suggesting the test is not suitable for this data. However, the Bayesian information criterion (BIC) value provides consistent estimates (Grasa, 1989) and the BIC value of the DINA model with hierarchical relations is smaller than that of the DINA model, which indicates a better fit of DINA model with hierarchical relations (see Supplementary Appendix Table A.3). The DINA model with hierarchical relations is also consistent with the hypothesis in the learning progression. Thus, we used the DINA model with hierarchical relations for subsequent analysis. We assume the ordering of the attributes involved in the learning progression of energy is deterministic, that is, mastery of strand 1’s attributes is prerequisite for mastery of strand 2’s attributes. It should be noted here that the higher-order DINA model (HO-DINA) is different from hierarchical CDMs. HO-DINA model refers to a higher-order latent trait in conjunction with the DINA model (De la Torre and Douglas, 2004; De la Torre, 2009). The higher-order latent trait can be interpreted as “a broadly defined general proficiency or overall aptitude in a particular domain” (De la Torre, 2009, p. 120). Students with higher proficiency are more likely to have a greater likelihood of mastering skills in this domain (De la Torre, 2009). Hierarchical CDMs constrain the number of permissible skills patterns using theoretically based hierarchical skills structures (De la Torre, 2009).

Second, we analyzed the achievement test items of each jurisdiction using the DINA model with hierarchical relations to obtain students’ mastery patterns. Third, we compared the similarities and differences between students’ mastery patterns in different jurisdictions from step one. Fourth, we analyzed how the intended curriculum may influence students’ mastery or understanding of the concept of energy. We used logistic regression to see whether students’ performance on each item differs depending on whether it was covered or not covered in the national curriculum, that is, detecting the instructional sensitivity of each item, using Mplus 8.5 software. In this step, the nested structure of the data (i.e., the class is nested within school) will be accounted for by using complex sampling adjustment analysis, that is, specifying the sampling probability weights and the class as a source of the clustering (Stapleton, 2006). The missing data will be handled through full information maximum likelihood estimation.

### DINA model

CDMs are special cases of latent class models that characterize the relationship of observable data to a set of categorical latent ability attributes (Templin and Henson, 2006). CDMs can diagnose the presence or absence of each attribute for every student and illuminate different mastery patterns. The DINA model is one of the most parsimonious and interpretable CDM models with only two item parameters (i.e., guessing parameter and slipping parameter). The DINA model is a noncompensatory model with a conjunctive condensation rule. The respondent needs to master all the attributes required for a particular item (Rupp et al., 2010) to have a high probability of answering the item correctly. A latent variable $\eta_{ij}$ represents whether or not respondent *i* has all of the required attributes to resolve item *j* in the DINA model (Hsu and Wang, 2015). The latent variable $\eta_{ij}$ is a function of the determinist input which is defined as *Equation 1*:


(1) $$\;\eta_{\mathrm{ij}}=\prod_{k=1}^{K}\alpha_{ik}^{q_{ik}}$$

where $\eta_{ij}$ = 1 when respondent *i* masters all of the required attributes for item *j,* $\eta_{ij}$ = 0 when respondent *i* lacks at least one of the required attributes, $\mathrm{and}\;\alpha_{ik}$ is the attribute vector for respondent *i* and attribute *k*. If an attribute is not measured by an item, then $q_{ik}$ = 0, which means that $\alpha_{jk}^{0}$ =1. If an attribute is measured by an item, then $q_{ik}$ = 1, which means that whether the respondent masters the attribute or not matters for the probability of correct response (Rupp et al., 2010).

The DINA model accounts for the noise (i.e., random error) introduced in the underlying stochastic process with *slip* and *guessing* parameters. Even respondents who have mastered all measured attributes for an item can slip and miss the item. The respondents who have not mastered at least one of the measured attributes may guess and answer a question correctly (Rupp et al., 2010). The probability of respondent *i* with the skill vector $\alpha_{i}$ answering item *j* correctly in the DINA model is defined as *Equation 2*:


(2) $$\;P\left(X_{ij}=1|\alpha_{i}\right)=g_{j}^{1-\eta_{ij}}{\left(1-s_{j}\right)}^{\eta_{ij}}$$

where $g_{j}$ is the guessing parameter, $s_{j}$ is the slipping parameter, and all other terms are as defined previously.

### Logistic regression

In this study, we used logistic regression to detect items’ instructional sensitivity. The outcome variable *Y_ij_* indicates the natural log odds of a correct response for student *i* on item *j*. The coding of items is put forward in the *variables* section above. Whether the item is covered in the national curriculum will be the predictor variable ( $Curriculum_{ij}$ ). The items covered in the national curriculum will be coded as 1; otherwise, they will be coded as 0. Studies exploring items’ instructional sensitivity using observational data control for students’ ability because students are not randomly assigned to instructional conditions (e.g., D’Agostino et al., 2007; Li et al., 2017). The rationale is that students’ ability would relate to students’ performance in each item, while performance on an instructional sensitive item is expected to increase with effective teaching (Baker, 1994). Thus, we examined the instructional sensitivity of the selected items, controlling for students’ ability ( $Ability_{ij}$ ). Instructional sensitivity analysis combines information from multiple attributes into a single ability score estimate. Students’ single ability is indicated by the number of attributes each student mastered from CDM analysis. The equation for the instructional sensitivity analysis, controlling students’ ability, is defined as *Equation 3:*


(3) $$\;Y_{ij}=\beta_{0j}+\beta_{1j}Curriculum_{ij}+\beta_{2j}Ability_{ij}\;$$

where $Y_{ij}$ is the log odds of a correct response for student *i* on item *j*, $\beta_{0j}$ is the log odds when the predictor variables are zero, and $\beta_{1j}$ is the logistic regression coefficient indicating instructional sensitivity regarding curriculum.

### Development of the draft Q-matrix and expert review

A well-designed Q-matrix is essential in CDMs. We developed a draft Q-matrix based on the literature related to learning theory, learning progressions of energy in the physical science domain (Neumann et al., 2013; Lacy et al., 2014), TIMSS assessment framework (Mullis et al., 2009), and Quebec *Progression of Learning Science and Technology* [Quebec Education Program (QEP), 2009]. There are two attributes under the “forms of energy” strand: Attribute 1 (A1) describes different forms of energy (mechanical, electrical, light, chemical, heat, sound, and nuclear); and A2 identifies sources of energy in his/her environment (e.g., moving water, the chemical reaction in a battery, and sunlight). There are four attributes under the “transfer and transformations of energy” strand: A3 distinguishes between substances that are conductors and those that are insulators; A4 explains that simple electrical systems, such as a flashlight, require a complete (unbroken) electrical pathway; A5 relates familiar physical phenomena to the behavior of light (e.g., reflections, rainbows, and shadows); and A6 understands heat transfer. According to the hypothesized learning progression from Lacy et al. (2014) and Neumann et al. (2013), we hypothesized that the six attributes in this study are not necessarily fully ordered, but the four attributes for the strand “transfer and transformations of energy” are followed by the two attributes for the strand “forms of energy” in learning sequence, and attributes are not ordered within the strands. There are 28 items in the proposed Q-matrix, and 24 out of 28 items (85.71%) are only measuring one attribute.

Then, five experts from science education were invited to review the draft matrix and the proposed attributes. The five experts were K-9 physical science teachers and faculty members in physical science education. All five experts had obtained their Bachelor’s degrees and Master’s degrees in science education. An interview was conducted with each expert by discussing each item’s endorsed attributes. The length of each interview was about an hour to an hour and a half. Experts were asked whether each endorsed attribute was correct or not, and what revisions needed to be made. Experts were also asked whether new attributes needed to be added to fully describe the available items’ content. The Q-matrix was revised after the expert review. Due to the word count limitation, the specific changes of Q-matrix would not be presented from expert review results in the main text but are listed in Supplementary Appendix B.

### Q-matrix validation using real data

The revised Q-matrix was analyzed and validated using CDMs and two split datasets. The current dataset was divided into halves. We randomly drew half of the data within each jurisdiction and combined those into one dataset for the first validation of the Q-matrix. We combined the rest of each jurisdiction’s data for the second validation. We conducted the validation analysis using the DINA model. We revised the Q-matrix according to the first analysis result, again referring to the expert review information. Then, we used the second half of the combined dataset to do the second validation analysis. Because the Q-matrix constructed by the domain experts may have misspecification, De la Torre and Chiu (2016) proposed a discrimination index, along with other indices to provide a more holistic approach to the diagnosis of model misfit (De la Torre and Chiu, 2016). In this study, we followed Mirzaei et al. (2020) procedure of diagnosing the model misfit. The absolute model fit will be identified using the standardized mean square root of squared residuals (SRMSR), mean of absolute deviations in observed and expected correlations (MADcor), mean of absolute values of Q3 statistic (MADQ3), and a maximum of all chi-square statistics (max[*χ*^2^]). To calculate MADQ3, residuals $\varepsilon_{ni}$ = $X_{ni}$ − $e_{ni}$ of observed and expected responses for respondents *n* and items *i* are constructed (Robitzsch et al., 2022, p. 167). Then, the average of the absolute values of pairwise correlations of these residuals is computed for MADQ3 (Robitzsch et al., 2022, p. 167). The max(*χ*^2^) statistic is the maximum of all item pairs *χ*^2^*_jj_* statistics, and a statistically significant *p*-value shows that some item pairs violate statistical independence (Robitzsch et al., 2022). Thus, a non-significant value for max(*χ*^2^) (*p* > 0.05) indicates a good fit. The reported *p*-value of max (*χ*^2^) is based on the Holm correction for multiple comparisons. For all other model fit indices, the model fits the data better if these fit indices are close to zero (Ravand and Robitzsch, 2015).

Item level fit will be evaluated using the item-fit root mean square error of approximation (RMSEA) and item discrimination index (IDI). The criteria for interpreting item-fit RMSEA are as follows: Item-fit RMSEA below 0.05 indicates good fit, and item-fit RMSEA below 0.10 indicates moderate fit (Kunina-Habenicht et al., 2009). IDI for each item is calculated as $IDI_{j}$ = $\;1-s_{j}-g_{j}$ (De la Torre, 2007; cited in Lee et al., 2012), where $\;s_{j}$ is the slipping parameter and $g_{j}$ is the guessing parameter. IDI can be used as a diagnostic index about how an item discriminates between students having a response probability of $1-s_{j}$ possessing all skills, and students guessing with probability ( $g_{j}$ ) without possessing any skills (George et al., 2016). IDI values close to 1 indicate good discrimination of the item, and IDI values close to 0 indicate items with low discrimination (George et al., 2016). The Q-matrix was revised according to the analysis result.

## Results

### Q-matrix validation results: Using real data

We divided the current data into two datasets, and we did the validation based on the first half of the data first using following procedure. First, we examined item-level fit indices to check how well the model fits each item’s observed response data. Item discrimination indexes (IDIs) are all good ranging from 0.103 to 0.883 except item S041191 had a negative IDI -0.002, which violated the assumption of the DINA model that $g_{j}$ < 1 − $s_{j}\;$ (George et al., 2016). Item S041191 was a multiple-choice item inquiring which material was the best conductor of heat. Then, we double-checked this item’s attribute classification (the endorsed attribute was A3 “Distinguishes between substances that are conductors and those that are insulators”) and consulted with the experts again, who indicated that no further changes of this item’s attribute should be made based on its content. Since there were still multiple items measuring A3, this item was deleted due to its negative IDI. All other item-level indices were good. The IDI indices ranged from 0.103 to 0.884. Item S051121A and item S051188E were two items with the lowest IDI index 0.103 and 0.137. Item S051121A and item S051188E were also found to have local dependence with other items (as presented in the next paragraph). Thus, these two items would be deleted. All items’ RMSEA values were below 0.05.

Then, we checked the absolute model fit indices. All other absolute model fit indices were good: SRMSR = 0.056, MADcor = 0.043, and MADQ3 = 0.080. However, the max (*χ*^2^) statistic was not good: max (*χ*^2^) = 38.029, *p* < 0.05. A significant *p*-value indicated a violation of the statistical independence of the item pair. Then, we checked the item pairs’ local independence. We found local dependence among multiple items, and most of them came from the same set of items. Also, two of these items had the lowest IDI among all the items. Thus, we deleted items that had largest chi-square statistics, items had statistically significant local dependence with other items, and/or items with a lower IDI, that is, S031273, S051201, S051121A, S051121B, S051188A, S051188B, and S051188E.

Then, we used the second half of the data to check the revised Q-matrix. The absolute model fit indices were all good: SRMSR = 0.034, MADcor = 0.024, MADQ3 = 0.084, max (*χ*^2^) = 9.485, and *p* = 0.052. The item-level fits were also all good. The IDI ranged from 0.104 to 0.942. RMSEA statistics were all below 0.05. Most items’ IDI increased at the second validation. We also double-checked the model fit and the item-fit indices for subset datasets for three jurisdictions. The absolute model fit indices (see Supplementary Appendix Table A.4) and item-fit indices were good for each jurisdiction (item-level RMSEA statistics were all below 0.05; IDI values were all above zero). We invited science experts to review the revised Q-matrix and check whether they endorse the attributes believed to be measured by each item. The experts confirmed the final Q-matrix. Table 1 presents the final Q-matrix of this study. In total, there are 16 items and six attributes in the final matrix.

**Table 1:** Final Q-matrix.

Items	A1	A2	A3	A4	A5	A6
S031077	0	0	1	0	0	0
S031197A	1	0	0	0	0	0
S031197B	1	0	0	0	0	0
S031298	0	0	0	0	0	1
S031299	1	0	0	0	1	0
S041067	1	0	0	0	0	0
S041069	0	0	0	0	1	0
S041070	0	0	0	0	1	0
S041195	0	0	0	1	0	0
S051074	0	0	0	1	0	0
S051179	0	0	0	0	1	0
S051121C	0	0	1	0	0	0
S051121D	0	0	1	0	0	0
S051121E	0	0	1	0	0	0
S051188C	0	1	0	0	0	0
S051188D	0	1	0	0	0	0

*Note.* A1 = Describes different forms of energy (mechanical, electrical, light, chemical, heat, sound, nuclear); A2 = Identifies sources of energy (e.g., moving water, the chemical reaction in a battery, sunlight); A3 = Distinguishes between substances that are conductors and those that are insulators; A4 = Explains that simple electrical systems, such as a flashlight, require a complete (unbroken) electrical pathway; A5 = Relates familiar physical phenomena to the behavior of light (e.g., reflections, rainbows, shadows); A6 = Recognizes that heating an object can increase its temperature and that hot objects can heat up cold objects.

### Attribute mastery profile across three jurisdictions

The population’s mastery probabilities are informative for the learning progressions (Briggs and Alonzo, 2012). Table 2 presents the attribute mastery probabilities of the three jurisdictions from DINA model’s analysis result. They show each participant population’s mastery probability for each attribute, which is the relative difficulty levels of different sub-skills underlying the energy topic for each jurisdiction. Attribute 1 (A1) “describes different forms of energy” and A2 “identifies sources of energy” from Strand 1 of the hypothesized learning progression had the highest mastery probabilities for Australia and Ontario. The highest mastery probability of the two attributes from Strand 1 indicates that the hypothesized learning progression could be matched to students’ observed progressions in understanding the energy concept using cognitive diagnostic models by detecting the attribute mastery probability. A4 “explains that simple electrical systems, such as a flashlight, require a complete (unbroken) electrical pathway” has the lowest mastery probability and was the most difficult for all students.

**Table 2:** Attribute mastery probabilities across three jurisdictions.

Attribute	Attribute mastery probability		
	Australia	Hong Kong	Ontario
A1	0.8068	0.9266	0.8583
A2	0.7332	0.9276	0.9203
A3	0.5947	0.8676	0.5482
A4	0.3517	0.4126	0.3637
A5	0.3806	0.4976	0.5314
A6	0.6975	0.3642	0.3968

### Latent class profiles

The latent class profiles could also inform the learning progressions. In this study, the DINA model with hierarchical relations defines 19 possible latent classes. Table 3 presents the latent class profiles and their attribute mastery pattern probabilities for each jurisdiction.

**Table 3:** Latent class probabilities.

Latent Class	Attribute Mastery Pattern	Australia	Hong Kong	Ontario
1	000000	0.05118	0.06194	0.05905
2	100000	0.04110	0.01042	0.02065
3	010000	0.05666	0.01143	0.08262
4	110000	0.04566	0.00190	0.02908
5	111000	0.07249	0.11679	0.06823
6	110100	0.03065	0.00190	0.02543
7	111100	0.05147	0.12521	0.06267
8	110010	0.02857	0.00296	0.04605
9	111010	0.10477	0.18201	0.10360
10	110110	0.02330	0.00178	0.03121
11	111110	0.09056	0.11949	0.07466
12	110001	0.03740	0.00958	0.02541
13	111001	0.04382	0.07425	0.03858
14	110101	0.02477	0.00956	0.02194
15	111101	0.03065	0.07943	0.03495
16	110011	0.03917	0.01309	0.06619
17	111011	0.10599	0.10307	0.09685
18	110111	0.03151	0.00787	0.04420
19	111111	0.09028	0.06731	0.06863

As is presented in Table 3, for Australia, the latent class “111011” had the highest latent class probability (0.10599 which means about 10.60% of the overall test-takers were estimated to have mastered all attributes). The latent class “111111,” to which 9.03% of the test-takers belong, came second. About 9.03% of students could not master A4 “explains that simple electrical systems” while they could master all other attributes. The third frequently mastered latent class is “111110” (0.09056), which means that about 9.056% of test-takers did not master A6 “recognizes that heating an object can increase its temperature and that hot objects can heat up cold objects” while they could master all other attributes. Besides, about 5.12% of test-takers did not master any attribute.

For Hong Kong, the highest probability is class “111010” and about 18.2% of test-takers were estimated to have mastered all attributes. The second-highest class probability of Hong Kong was also a latent class “111100” (0.1252). About 12.52% of Hong Kong students could not master A4 “explains that simple electrical systems,” while they could master all other attributes. The percentage was relatively higher than for Australian students. The third highest class probability is “111110,” with about 11.95% of test-takers possessing this latent class.

For Ontario, the highest probability is class “111010” and about 10.36% of test-takers did not master all the attributes except A4 (i.e., explains that simple electrical systems, such as a flashlight, require a complete electrical pathway) and A5 (i.e., relates familiar physical phenomena to the behavior of light). The second-highest class probability of Ontario is the latent class “111011” (0.09685), which means that about 9.685% of test-takers are masters of all the attributes. The third highest class probability is “010000,” with about 8.26% of test-takers only mastering A2 “identifies sources of energy.”

### Instructional sensitivity of the selected items after controlling student ability

From TCMA data, we obtained the results of whether the selected items in this study were covered in the selected jurisdiction’s national curriculum or not (see Table 4). Of 16 items, 6 (i.e., S031077, S031299, S041069, S041070, S041195, and S051179) did not have variation in national curriculum coverage. Items without variation will not provide any information about instructional sensitivity. Thus, these items were dropped. Then, we examined the instructional sensitivity of all items with variation in national curricular coverage, controlling for students’ abilities. We calculated the number of attributes each student mastered and treated this as an estimate of their overall competence in the energy domain. Table 5 presents results, and eight items were found to be instructionally sensitive, that is, students have a better understanding of the item if the item is covered in the curriculum. Item S051074 assessing the description of forms of energy has the largest regression coefficient, 4.157, which indicates students whose curriculum covered the item have 63.88 times greater odds (*e*^4.157^) of scoring in a higher response category than students whose curriculum did not cover it. Item S051121C assessing whether students can distinguish between substances that are conductors and those that are insulators has a relatively high regression coefficient, 2.747, which means students whose curriculum covered the item have 15.60 times greater odds (*e*^2.747^) of scoring in higher response category than those not.

**Table 4:** Items covered in national curricula.

Items	Item covered in national curriculum		
	Australia	Hong Kong	Ontario
S031077	no	no	no
S031197A	yes	no	no
S031197B	yes	no	no
S031298	yes	yes	no
S031299	yes	yes	yes
S041067	yes	no	no
S041069	yes	yes	yes
S041070	yes	yes	yes
S041195	no	no	no
S051074	no	yes	no
S051179	yes	yes	yes
S051121C	yes	yes	no
S051121D	yes	yes	no
S051121E	yes	yes	no
S051188C	yes	yes	no
S051188D	yes	yes	no

*Note.* This data is from the TIMSS Test Curriculum Matching Analysis (IEA, 2013).

**Table 5:** Results of the instructional sensitivity for all items after controlling student ability.

Items	Logistic regression coefficient of curriculum	*e* ^coefficient^	*p*
S031197A*	1.411	4.10	0.045
S031197B*	1.585	4.88	0.008
S030298*	1.878	6.54	0.000
S041067*	0.477	1.61	0.000
S051074*	4.157	63.88	0.000
S051121C*	2.747	15.60	0.000
S051121D*	2.090	8.08	0.000
S051121E*	2.060	7.85	0.001
S051188C	0.745	2.11	0.143
S051188D	1.097	3.00	0.755

*Note*. * Items that were found to show instructional sensitivity.

*e*^coefficient^ stands for *e* to the power of logistic regression coefficient of curriculum, where *e* is 2.71828.

## Discussion

### Students’ learning progressions in energy across three jurisdictions

The highest mastery probability of the two attributes from Strand 1 indicates that the hypothesized learning progression could be matched to students’ observed progressions in understanding the energy concept using cognitive diagnostic models by detecting the attribute mastery probability. This is consistent with previous research about the learning progression in energy (Neumann et al., 2013; Lacy et al., 2014) that showed the stand “forms of energy” learned before the Strand “transfer and transformations of energy” in the learning progression. In addition, we also found that A4 from Strand 2 was learned the latest by students. The mastery probability of the A4 “explains that simple electrical systems, such as a flashlight, require a complete electrical pathway” from Strand 2 “transfer and transformation of energy” is the lowest among all the attributes for all the three selected participating jurisdictions: Australia (0.3517), Hong Kong (0.4126), and Ontario (0.3637). Besides, almost 10% of students from each jurisdiction (Australia, 10.6%; Hong Kong, 10.3%; and Ontario, 9.68%) have the latent class pattern (111011). These results show that no matter where students came from, they performed worse in mastering A4 “explains that simple electrical systems, such as a flashlight, require a complete electrical pathway,” and more than half of the students in each jurisdiction failed to acquire A4. There were mainly two items assessing A4: item S041195 and item S051074. For item S041195, none of the three jurisdictions’ curriculum covered this item. Item S051074 showed large instructional sensitivity, which means that the performance of the item was related to whether the item was covered in the curriculum or not. Students performed better at this item if this item was covered in the national curriculum. However, neither Ontario nor Australia covered this item in the national curriculum. When we examined the description of energy for each jurisdiction in the curriculum carefully, “electrical circuits” [Australian Curriculum, Assessment and Reporting Authority (ACARA), 2020] were highlighted in Australia’s curriculum in Grade 6. Similarly, the Ontario curriculum described “simple circuits” in Grade 6. Though Hong Kong reported covering this item in their curriculum, the grade band structure of the national curriculum makes it difficult to identify whether circuits are generally covered in Grades 4, 5, and/or 6. Thus, there was still a large possibility that students in Grade 4 had not had the opportunity to learn to master this attribute. In addition, students’ misconceptions about circuits are common worldwide (Moodley and Gaigher, 2019). This may explain students’ lowest mastery of Attribute 4 “explains that simple electrical systems, such as a flashlight, require a complete electrical pathway” in this study. Studies have shown that students have many different misconceptions about electric circuits (e.g., Çepni and Keleş, 2006; Peşman and Eryılmaz, 2010). For instance, Çepni and Keleş (2006) summarized four models used by students that resulted in misunderstanding circuits: a unipolar model; the clashing current model; the current consumed model; and the scientist model with current conserved. For example, in the unipolar model, students believe that only one cable is enough to complete a circuit, which would hinder their mastery of A4 “explains that simple electrical systems, such as a flashlight, require a complete electrical pathway.” Science teachers should get to know different misconceptions that students have in mastering A4 and utilize these misconceptions to help students to change their misconceptions and enhance their conceptual understanding, for example, by asking students to demonstrate that one cable is not enough to complete a circuit.

The content of a country’s curriculum (i.e., the intended curriculum) has been shown to affect students’ performance (Schmidt et al., 2001; Ramírez, 2006). Schmidt et al. (2005) also found that curricular coherence was the most dominant predictive factor for Grade 1 to Grade 8 students’ academic performance in science and mathematics, where the curricular coherence is defined as curriculum standards sequenced progressively toward the understanding of the deeper structure of each topic both within and across grades. This study reemphasized the importance of the curriculum for students’ performance, which is consistent with earlier studies (Schmidt et al., 2001, 2005; Ramírez, 2006). In addition, LPs can provide a framework to coordinate standards, assessments, and instruction (Alonzo and Gotwals, 2012). The alignment of standards, assessments, and instruction could be achieved through LPs. LPs are essential in designing curricula materials that allow learners to develop integrated understandings of key scientific ideas and practices across time (Fortus and Krajcik, 2012). However, currently, not all curricula are designed based on students’ LPs. It is common that the curriculum was not built to coherently help learners make connections between ideas within and among disciplines nor help learners develop an integrated understanding (Fortus and Krajcik, 2012). The development of coherent curriculum materials calls for “multiple cycles of design and development, testing and revising the materials, aligning materials, assessments, and teacher support with learning progressions” (Fortus and Krajcik, 2012, p. 796).

### Students’ knowledge mastery patterns for different jurisdiction

Overall, this study’s results showed that Australia had the highest percentages of students mastering all the attributes, while lower percentages of Ontario students mastered all the attributes and most individual attributes. These indicate that Ontario students perform relatively worse than Australian and Hong Kong students on the energy topic. Among 16 selected items assessing the attributes in the Q-matrix, Hong Kong had 11 items that were reported to be covered in their curriculum according to the TCMA data and Australia had 13 items. However, Ontario only had four items, many fewer than were covered in the comparison jurisdictions. Ontario students’ relatively poor performance in energy learning may be caused by their much lesser curriculum exposure to learn these attributes.

This study found some other similarities in students’ knowledge mastery patterns across the selected jurisdictions using cognitive diagnostic models. There were high proportions of students in the latent class pattern “111010” for all three jurisdictions. This implicated that most students had weakness in mastering both A4 “explains that simple electrical systems, such as a flashlight, require a complete electrical pathway” and A6 “recognizes that heating an object can increase its temperature and that hot objects can heat up cold objects.” The latent class pattern “111110” was another frequent mastered knowledge mastery pattern among three jurisdictions: Besides, the overall mastery probability of A6 was the second-lowest next to A4’s. These results indicated that A6 “recognizes that heating an object can increase its temperature and that hot objects can heat up cold objects” was also difficult for all the participants from three jurisdictions. Students in primary schools always hold some misconceptions about heat and temperature. Students may believe the temperature of an object is related to its physical properties, that is, the object’s temperature differs by its material properties (Erickson and Tiberghien, 1985; Paik et al., 2007), and may confuse it with heat (Paik et al., 2007). For instance, some students in primary schools thought that objects of different materials in the same room were at different temperatures, and there was a misconception of the students that wood objects were hotter than metal objects (Erickson and Tiberghien, 1985). These misconceptions about the temperature of objects may lead to students’ poor mastery in A6.

### Limitations and future directions

Since this study used existing TIMSS Grade 4 science datasets, we could only detect students’ proficiency on attributes of the energy topic that were measured by the test’s items, and only two strands of the hypothesized learning progression could be tested due to the limited number of items that TIMSS administered on the energy topic. In future research, we could develop an assessment from a cognitive diagnostic model approach to include more attributes, so we can separately detect more abilities and skills of students’ energy mastery learning progression (Neumann et al., 2013). Second, in the Q-matrix validation process, we invited experts in physical science education to review the Q-matrix while Grade 4 students were not interviewed to talk through their problem-solving methods for each item. In future research, we could also include students’ think-aloud process for each item to validate the Q-matrix. It would be more comprehensive to include both experts’ and students’ views. Third, we coded all students’ responses as binary and applied a CDM for dichotomous item responses. This recoding would be expected to result in some loss of information, compared to using the original polytomous responses. A diagnostic model for ordinal polytomous data has been formulated by Ma and de la Torre (2016) and Culpepper (2019), but is not yet implemented in widely available software. In future studies, researchers may be able to fit polytomous CDMs to detect students’ LPs. Fourth, we attempted to run multiple-group CDM for detecting differential item functioning (DIF) across the three countries. We tried DIF procedures [i.e., Wald test (Hou et al., 2014)], but the codes only work for the DINA model and cannot work for the DINA model with hierarchical relations, which results in warnings and the code cannot be run. There may be some items that function differentially across three countries but could not be identified based on this study. Future studies could work on detecting DIF using a multiple-group DINA model with hierarchical relations. Fifth, this study mainly considered whether the item is covered in the curriculum as reported in the TIMSS TCMA data as the potential factor contributing to students’ performance of each item in the analysis. In future studies, we could test other contextual and cultural factors. Finally, this study only included three jurisdictions in the analysis. We will extend the analysis to more jurisdictions in future. In summary, our study indicates that CDM can be used to validate students’ learning progression and curriculum-related issues may influence students’ learning progression. Future studies could explore other domain’s learning progressions using CDM.

## Data availability statement

The original contributions presented in the study are included in the article/Supplementary material, further inquiries can be directed to the corresponding author.

## Author contributions

SZ wrote the introduction, literature review, did the analysis, wrote up method, results, and discussion parts. AT gave feedbacks and revised the paper. All authors contributed to the article and approved the submitted version.

## Funding

The research is supported by the Fundamental Research Funds for the Central Universities (No. 2232022E-15). The work reported here is based on the dissertation by the first author submitted in partial fulfillment of the requirements of the degree of Doctor of Philosophy at Purdue University.

## Conflict of interest

The authors declare that the research was conducted in the absence of any commercial or financial relationships that could be construed as a potential conflict of interest.

## Publisher’s note

All claims expressed in this article are solely those of the authors and do not necessarily represent those of their affiliated organizations, or those of the publisher, the editors and the reviewers. Any product that may be evaluated in this article, or claim that may be made by its manufacturer, is not guaranteed or endorsed by the publisher.

## Supplementary material

The supplementary material for this article can be found online at: https://www.frontiersin.org/articles/10.3389/fpsyg. 2022.892884/full#supplementary-material
