# Supplementary material for: Measuring students’ learning progressions in energy using cognitive diagnostic models
Source: Front Psychol. 2022 Aug 9;13:892884. doi: 10.3389/fpsyg.2022.892884 (PMC9396370; doi:10.3389/fpsyg.2022.892884)
Supplement: Supplementary file 1 [file Data_Sheet_1.docx]

**Appendices**

**Appendix A**

| Table A.1 | | | |
| --- | --- | --- | --- |
| *Selected Variable List of TIMSS 2011 Grade 4 Data* | | | |
| Variable | Cognitive Domain | Question type | Response Category |
| S031273 | Applying | Multiple choice | 4 |
| S031076 | Reasoning | Open-ended | 3 |
| S031077 | Applying | Multiple choice | 4 |
| S031197A | Knowing | Open-ended | 7 |
| S031197B | Knowing | Open-ended | 7 |
| S031298 | Applying | Multiple choice | 4 |
| S031299 | Knowing | Open-ended | 5 |
| S041311 | Applying | Multiple choice | 4 |
| S041120 | Knowing | Multiple choice | 4 |
| S041067 | Knowing | Open-ended | 2 |
| S041069 | Applying | Multiple choice | 4 |
| S041070 | Applying | Multiple choice | 4 |
| S041191 | Knowing | Multiple choice | 4 |
| S041195 | Applying | Open-ended | 3 |
| S051119 | Reasoning | Open-ended | 3 |
| S051074 | Applying | Open-ended | 3 |
| S051179 | Applying | Multiple choice | 4 |
| S051201 | Applying | Multiple choice | 2 |
| S051121A | Knowing | Multiple choice | 2 |
| S051121B | Knowing | Multiple choice | 2 |
| S051121C | Knowing | Multiple choice | 2 |
| S051121D | Knowing | Multiple choice | 2 |
| S051121E | Knowing | Multiple choice | 2 |
| S051188A | Knowing | Multiple choice | 2 |
| S051188B | Knowing | Multiple choice | 2 |
| S051188C | Knowing | Multiple choice | 2 |
| S051188D | Knowing | Multiple choice | 2 |
| S051188E | Knowing | Multiple choice | 2 |

| Table A.2 | | | | | | |
| --- | --- | --- | --- | --- | --- | --- |
| *Proposed Q matrix* | | | | | | |
| Items | A1 | A2 | A3 | A4 | A5 | A6 |
| S031273 | 0 | 0 | 1 | 0 | 0 | 1 |
| S031076 | 0 | 1 | 0 | 0 | 0 | 0 |
| S031077 | 0 | 1 | 1 | 0 | 0 | 0 |
| S031197A | 1 | 0 | 0 | 0 | 0 | 0 |
| S031197B | 1 | 0 | 0 | 0 | 0 | 0 |
| S031298 | 0 | 1 | 0 | 0 | 0 | 1 |
| S031299 | 1 | 0 | 0 | 0 | 0 | 0 |
| S041311 | 1 | 0 | 0 | 0 | 0 | 1 |
| S041120 | 0 | 1 | 0 | 0 | 0 | 0 |
| S041067 | 1 | 1 | 0 | 0 | 0 | 0 |
| S041069 | 0 | 0 | 0 | 0 | 1 | 0 |
| S041070 | 0 | 0 | 0 | 0 | 1 | 0 |
| S041191 | 0 | 0 | 1 | 0 | 0 | 0 |
| S041195 | 0 | 0 | 0 | 1 | 0 | 0 |
| S051119 | 0 | 1 | 0 | 0 | 0 | 0 |
| S051074 | 0 | 0 | 0 | 1 | 0 | 0 |
| S051179 | 0 | 0 | 0 | 0 | 1 | 0 |
| S051201 | 0 | 0 | 1 | 0 | 0 | 0 |
| S051121A | 0 | 0 | 1 | 0 | 0 | 0 |
| S051121B | 0 | 0 | 1 | 0 | 0 | 0 |
| S051121C | 0 | 0 | 1 | 0 | 0 | 0 |
| S051121D | 0 | 0 | 1 | 0 | 0 | 0 |
| S051121E | 0 | 0 | 1 | 0 | 0 | 0 |
| S051188A | 0 | 1 | 0 | 0 | 0 | 0 |
| S051188B | 0 | 1 | 0 | 0 | 0 | 0 |
| S051188C | 0 | 1 | 0 | 0 | 0 | 0 |
| S051188D | 0 | 1 | 0 | 0 | 0 | 0 |
| S051188E | 0 | 1 | 0 | 0 | 0 | 0 |
| *0te.* A1 = Describes different forms of energy (mechanical, electrical, light, chemical, heat, sound, nuclear); A2 = Identifies sources of energy (e.g. moving water, the chemical reaction in a battery, sunlight); A3 = Distinguishes between substances that are conductors and those that are insulators; A4 = Explains that simple electrical systems, such as a flashlight, require a complete (unbroken) electrical pathway; A5 = Relates familiar physical phenomena to the behavior of light (e.g., reflections, rainbows, shadows); A6 = Understands heat transfer | | | | | | |

| Table A.3 |  |  |  |  |  |
| --- | --- | --- | --- | --- | --- |
| *Model Fit Statistics for Alternative Models* | | | | | |
| Model | Log-likelihood | Deviance | Number of Parameters | AIC | BIC |
| DINA with hierarchical relations | -12484.40 | 24968.80 | 66 | 25100.80 | 25556.21 |
| DINA | -12484.44 | 24968.87 | 70 | 25108.87 | 25591.88 |

| Table A.4 | | | | |
| --- | --- | --- | --- | --- |
| Absolute Model Fit Statistics for Australia, Hong Kong, and Ontario | | | | |
| Jurisdiction | max(χ^2^) | MADcor | SRMSR | MADQ3 |
| Australia | 6.446 (*p* = 0.278) | 0.028 | 0.037 | 0.094 |
| Hong Kong | 3.939 (*p* = 1.000) | 0.031 | 0.044 | 0.085 |
| Ontario | 3.088 (*p* = 1.000) | 0.035 | 0.045 | 0.081 |

| Table A.5 | | | | |
| --- | --- | --- | --- | --- |
| *Item Statistics: Proportion-correct Item Difficulty* | | | | |
| Item | Proportion-correct Item Difficulty | | | |
|  | Australia | Hong Kong | Ontario | |
| S031273 | 0.66 | 0.87 | 0.63 | |
| S031076 | 0.37 | 0.50 | 0.53 | |
| S031077 | 0.76 | 0.84 | 0.80 | |
| S031197A | 0.86 | 0.81 | 0.85 | |
| S031197B | 0.77 | 0.69 | 0.77 | |
| S031298 | 0.29 | 0.44 | 0.26 |  |
| S031299 | 0.45 | 0.54 | 0.57 | |
| S041311 | 0.94 | 0.96 | 0.94 | |
| S041120 | 0.45 | 0.26 | 0.47 | |
| S041067 | 0.65 | 0.66 | 0.63 | |
| S041069 | 0.60 | 0.73 | 0.57 | |
| S041070 | 0.63 | 0.51 | 0.62 | |
| S041195 | 0.14 | 0.21 | 0.20 | |
| S051119 | 0.26 | 0.32 | 0.39 | |
| S051074 | 0.18 | 0.23 | 0.12 | |
| S051179 | 0.85 | 0.74 | 0.88 | |
| S051201 | 0.55 | 0.17 | 0.55 | |
| S051121A | 0.84 | 0.89 | 0.90 | |
| S051121B | 0.82 | 0.84 | 0.78 | |
| S051121C | 0.76 | 0.89 | 0.69 | |
| S051121E | 0.72 | 0.94 | 0.72 | |
| S051188A | 0.84 | 0.89 | 0.89 | |
| S051188B | 0.75 | 0.89 | 0.77 | |
| S051188C | 0.93 | 0.93 | 0.95 | |
| S051188D | 0.90 | 0.93 | 0.94 | |
| S051188E | 0.69 | 0.92 | 0.75 | |

**Appendix B**

## **Q Matrix Validation Results: Expert Review**

We summarized the four experts’ feedback and revised the proposed Q matrix according to their feedback. One attribute’s description was revised, some items were deleted from the matrix, and some items’ attributes’ endorsement was changed. First, one attribute’s description was revised according to experts’ review. Experts commented that Attribute 6 was broad, and we narrowed down Attribute 6 “Understand heat transfer” to “Recognize that heating an object can increase its temperature and that hot objects can heat up cold objects” based on the content of TIMSS items.

Second, experts also suggested deleting some items. Item S031076 was about magnets repelling or attracting because of North/South poles repelling or attracting rather than about Attribute 2, “Identifies sources of energy.” Since only one item assessed the attribute about magnetism proposed by the reviewer, which could not provide adequate estimation, this item was deleted. There were still 9 items assessing Attribute 2 in the Q matrix after item S031076 was deleted, which would not affect the testing of the hypothesized learning progressions. Item S051119 was about the reasoning of magnetic property that magnets can attract pins and only this item measured this content. Thus, this item was also deleted. Although item S041311 was under the “source and effects of energy” topic area, it is about the reading of a thermometer and was not related to any attributes proposed. Since only one item was related to thermometer reading, this item was deleted. Item S041120 about the objects that produce their own light was also deleted since this item is not related to any attributes proposed.

Some items’ attribute endorsement changed according to experts’ review. For item S031077, Attribute 2 was not endorsed since all the experts agreed that this item did not involve identifying sources of energy as proposed in Attribute 2. For item S031298, Attribute 2 was also not endorsed since students did not need to identify sources of energy to solve this problem. For item S031299, Attribute 5 was added since it is about light rays as proposed in Attribute 5. For item S041067, Attribute 2 was deleted. For item S051201, it assessed whether students understand sweaters are insulators or not. Thus, Attribute 3 was endorsed, and Attribute 6 was not endorsed. In summary, four items were deleted (i.e., S031076, S051119, S041311, S041120) and there remained six attributes according to the expert review’s feedback. Eight items assessed the attributes of the first strand of the proposed learning progressions and seventeen items assessed the attributes of the second strand of the proposed learning progressions.
